# A Chromosomal Inversion of 46XX, inv (6) (p21.3p23) Connects to Congenital Heart Defects

**DOI:** 10.3389/fcvm.2020.00121

**Published:** 2020-07-31

**Authors:** Liangping Cheng, Yanlai Tang, Yuese Lin, Hongjun Ba, Yiqian Ding, Dubo Chen, Min Liu, Peizhen Pan, Youzhen Qin, Zhan-Peng Huang

**Affiliations:** ^1^Department of Cardiology, Center for Translational Medicine, Institute of Precision Medicine, The First Affiliated Hospital, Sun Yat-sen University, Guangzhou, China; ^2^Department of Pediatric Cardiology, Heart Center, The First Affiliated Hospital, Sun Yat-sen University, Guangzhou, China; ^3^NHC Key Laboratory of Assisted Circulation, Sun Yat-sen University, Guangzhou, China; ^4^Department of Pediatrics, The First Affiliated Hospital, Sun Yat-sen University, Guangzhou, China; ^5^Department of Laboratory Medicine, The First Affiliated Hospital, Sun Yat-sen University, Guangzhou, China

**Keywords:** congenital heart disease, ventricular septal defect, chromosomal rearrangement, human chromosome 6, proband

## Abstract

Congenital heart defects (CHDs) represent the most common human birth defects. Ventricular septal defect (VSD) is the most common subtype of CHDs. It has been shown that about 20–40% of VSDs are closely related to chromosomal aneuploidies or Mendelian diseases. In this study, we report a pedigree with VSD associated with a balanced paracentric inversion of chromosome 6, inv (6)(p21.3p23), a rarely reported CHD-associated chromosomal abnormality related to the fragile site at 6p23. We have found that the major clinical features of the proband include CHDs (ventricular septal defect, severe pulmonary hypertension, tricuspid regurgitation, and patent foramen ovale), severe pneumonia, and growth retardation. Our study reports a rare chromosomal abnormality connected to CHDs, which may represent a new genetic etiology for VSD.

## Introduction

Congenital heart defects (CHDs) represent the most common human birth defects, affecting about 1% newborns worldwide (1). Ventricular septal defect (VSD) is the most common form of CHDs. Around 30% of cases of CHDs are diagnosed after birth and 10% of all fetal cases are associated with VSD (2, 3). Data showed that ~20–40% of VSDs occurred due to chromosomal aneuploidies or Mendelian diseases, while the rest of the cases remained idiopathic (4–8). Furthermore, 33–47% of fetuses with VSD had chromosomal abnormalities, while trisomy 18, trisomy 21, and DiGeorge syndrome were the most common cases among these mutations (5, 7, 9, 10). The defect in the ventricular septum causes the leakage of blood from the left ventricle to the right ventricle of the heart. Instead of pumping out to the body, a portion of oxygen-rich blood pumps back to the lungs, which causes the heart to work harder. Meanwhile, pulmonary arteries thicken or grow rigid and become narrowed inside where the blood flows, and then pulmonary arterial hypertension (PAH) occurs (11). When PAH happens, the patient's body cannot get the oxygen it needs. As a result, he/she grows tired more easily. Other symptoms will also turn on, like shortness of breath, chest pain or pressure, heart palpitations, dizziness, fainting, swelling in their arms and legs, racing pulse, etc.

Defects in cardiac development often lead to congenital heart disease. It has been shown that both genetics and environmental factors affect the pathogenesis of CHDs (12, 13), but the underlying mechanism is still not fully understood. Ventricular septal defect may be inherited and sometimes is associated with other congenital disorders, such as Down syndrome. As the commonest congenital cardiac malformations (14), VSDs were widely studied. In many cases, VSD is not simply induced by a specific genetic problem, but genes probably play a role along with environmental factors. Some cases of VSD are passed from generation to generation, while non-genetic-related VSD is not inherited.

In this report, we describe a pedigree with congenital heart defects, including VSD, which carries a balanced paracentric inversion of chromosome 6, inv (6) (p21.3p23). Although a similar case was found in a pedigree with hereditary hemochromatosis (15), the chromosomal rearrangement related to the fragile site at 6p23 is rarely reported to be linked to congenital heart disease. Our study indicates that this reported chromosomal abnormality may represent a new genetic etiology for VSD.

## Materials and Methods

Genetic pedigrees are mapped with the Panogram software (https://github.com/panogram) through a detailed medical history inquiry. Panogram is an offline, stand-alone multiplatform pedigree drawing tool based on the Phenotips (https://github.com/phenotips/phenotips) platform. This platform included clinical symptoms and physical findings, family information and history (including pedigree), diagnosis (mapped to OMIM or Orphanet), genes and variants of interest, measurements (with support for the instant computation of percentiles and generation of growth charts), and demographic information (name, date of birth, etc.).

Standard phytohemagglutinin-stimulated lymphocyte chromosomes were prepared from peripheral blood lymphocytes from the patients. A karyotype analysis of the patients was performed by KingMed Diagnostics using GTG banding (Giemsa-trypsin) approach.

A clinical examination of the child was carried out. Chromosomal microarray analysis (CMA) was carried out to detect chromosomal imbalances and copy number variants in the proband. Echocardiogram and chest radiographs were carried out to detect disease progression in the proband. Her family members also had echocardiogram and detection of a series of biochemical indicators of ferritin according to the standard clinical protocol.

### Ethics Statement

All individuals involved in the study have signed an informed consent.

## The Proband Carries Multiple Congenital Heart Defects

**Figure 1:**
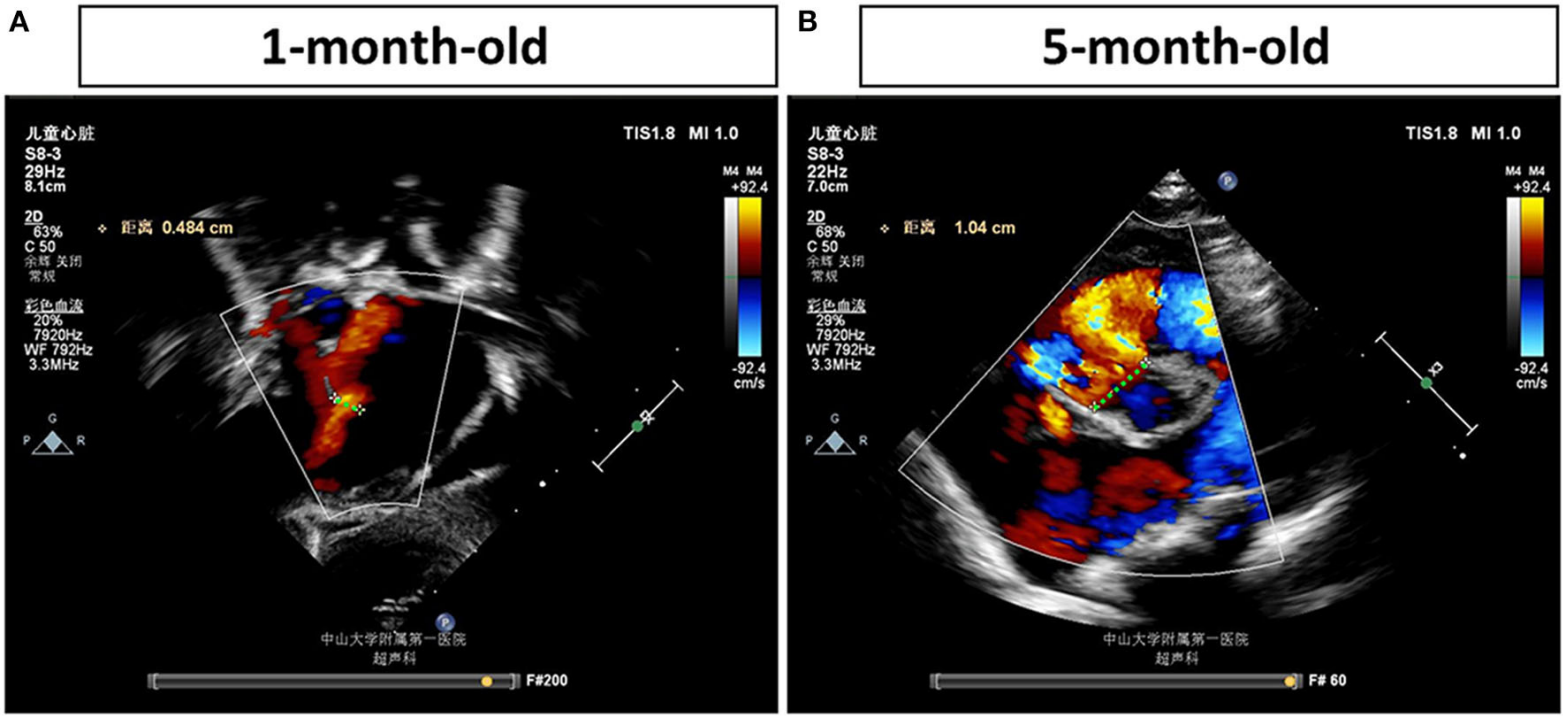
Echocardiogram examinations of the proband at different time points. Representative image of the echocardiogram of the proband's left ventricle obtained in the parasternal long-axis view **(A)** shows a ventricular septal defect (VSD) (0.484 cm) at 1 month of age. Representative image of the echocardiogram **(B)** shows a more severe VSD, which is enlarged to 1.04 cm, in the proband's heart at 5 months of age. The largest distances of VSD are marked by green dash lines and measured.

The proband is an 8-month-old baby girl (V-4) who came to our facility for chromosomal examination at 1 month of age since she was found to have a cardiac abnormality before she was born. She was born at full term by cesarean section and was admitted to the local hospital due to continued low blood oxygen saturation after birth. In our facility, blood samples were collected for CMA testing (based on the Affymetrix CytoScan HD array). However, no abnormalities in the chromosome copy number variants and absence of heterozygosity were found (data not shown). After 4 months, the proband was hospitalized in our facility due to a decrease in the amount of milk consumed. A heart ultrasound examination showed that she had large ventricular septal defect (perimembranous), atrial septal defect (secondary foramen), and severe pulmonary hypertension (Figure 1). Her weight gain was only 1.5 kg in 4 months (from 3.4 to 4.9 kg, ~40% increase). The VSD progressed from 0.484 to 1.04 cm in 4 months, as determined by echocardiogram (Figure 1), indicating that the defect becomes more severe (a larger percentage of VSD enlargement than her body growth). The proband was given cedar orchid digitalis, oral digoxin, and spironolactone, to adjust the heart function, and related symptomatic supportive treatment. Her milk consumption volume was gradually increased to 50–70 ml/q3h after treatment, and the patient was discharged. The proband was re-admitted to our facility at 6 months of age for a cardiac surgery. The surgery was successfully performed, which has repaired the interventional inferior ventricular septal defect in the proband.

## A VSD Pedigree With Chromosomal Inversion in Chromosome 6

**Figure 2:**
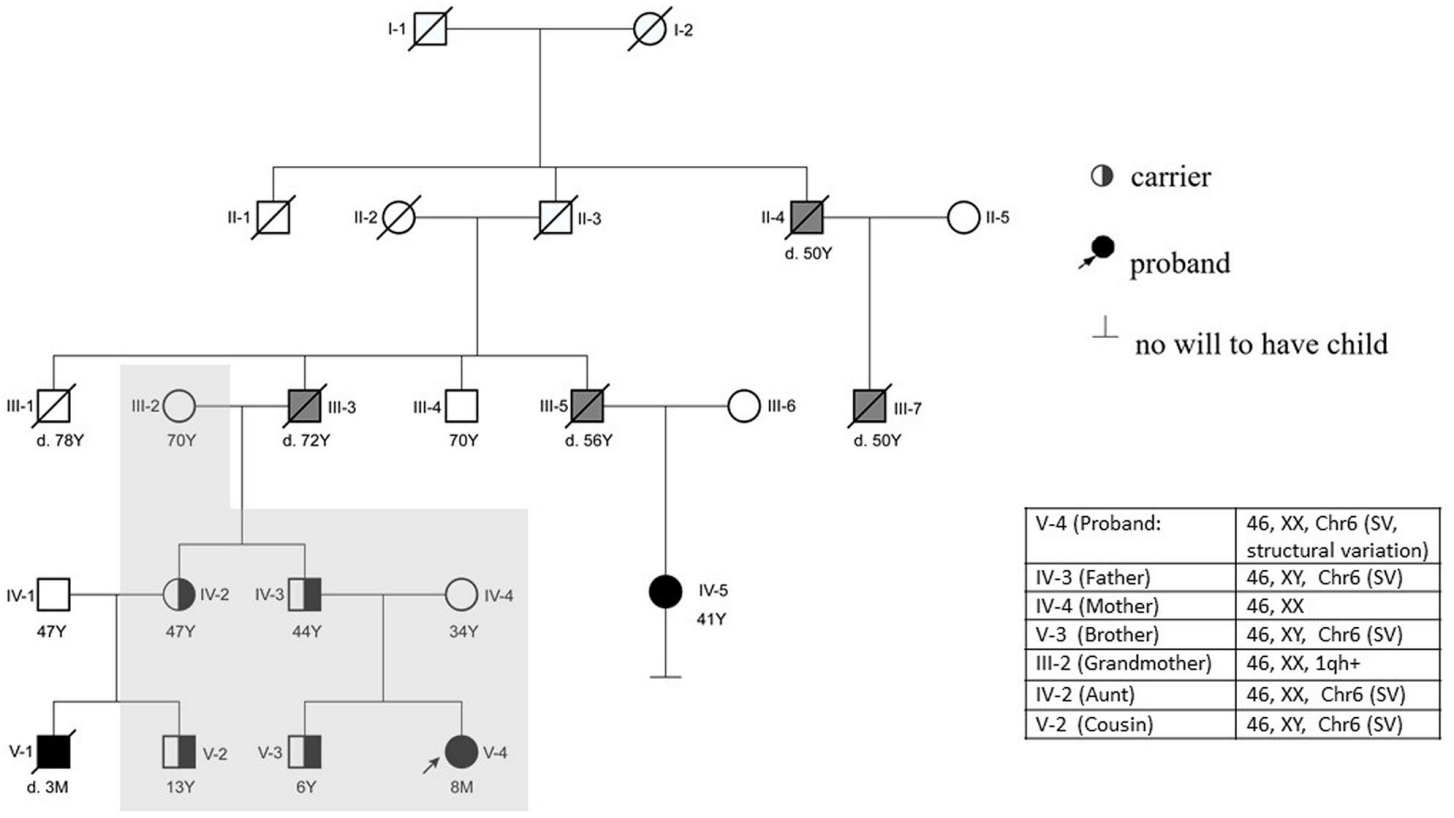
Pedigree of the reported family. The proband (V-4) is indicated with an arrow. The proband's mother (IV-4) has a normal phenotype. V-1, V-4, IV-5, III-3, III-5, III-7, and II-4 were reported to have cardiac abnormalities. The proband (V-4) had a surgery at 6 months. IV-5 and V-1 were found to suffer from VSD earlier than the proband. V-1 died at 90 days postnatal; IV-5 is alive after she had a surgery 30 years ago. The karyotype of the proband's father (IV-3), brother (V-3), aunt (IV-2), and cousin (V-2) are the same as the proband's karyotype. The proband's grandfather (III-3) and other relatives (II-4, III-5, and III-7) died of heart disease according to information provided by the proband's father (Y, years; M, month; d.3M, died at the age of 3 months). The individuals with a confirmed VSD are filled in black. The individuals reported to be having other cardiac diseases are filled in gray. The individuals who have done the karyotype examination in this study are indicated with a shadow.

**Figure 3:**
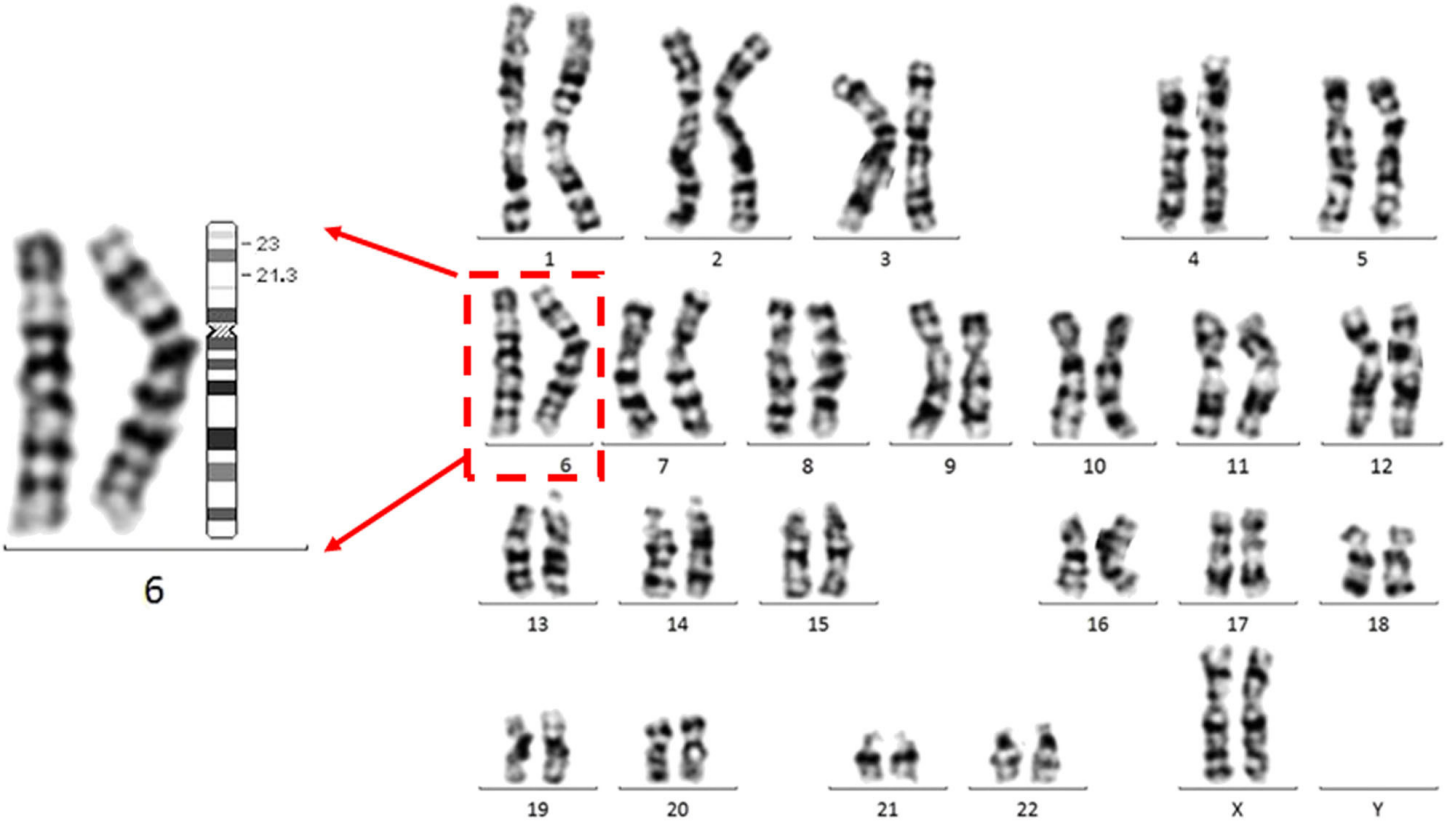
The proband carries a chromosomal inversion in chromosome 6.Representative G banded chromosomes from the proband. Enlarged chromosome 6 is shown at the left. The karyotype represents a p21.3 in chromosome 6 invasion with p23 <46, XX, inv (6) (p21.3p23)>. An ideogram of chromosome 6 is shown. The breakpoints are indicated in the ideogram.

A follow-up medical survey revealed that one of proband's aunts (IV-5) had undergone surgical correction for ventricular septal defect when she was an adolescent. Another biological aunt (IV-2) also had a child (V-1) with VSD 13 years ago and who died 3 months after birth (Figure 2). We speculated that the occurrence of VSD in this pedigree is related to genetic factors. Since no abnormality was shown in CMA testing, the proband's karyotype was further examined. As a result, a chromosomal inversion in chromosome 6<46, XX, inv (6) (p21.3p23)> was found (Figure 3). Furthermore, blood samples were collected from six other individuals in this pedigree, including the proband's grandmother (III-2), parents (IV-3 and IV-4), aunt (IV-2), elder brother (V-3), and cousin (V-2), for karyotype examination. The result indicated that the proband's father (IV-3), aunt (IV-2), elder brother (V-3), and cousin (V-2) carry the same mutation as the proband's mutation. The proband's grandmother (III-2) carries a polypeptide change on chromosome 1, which was not detected in the other members. No obvious VSD was found from their provided information nor from the on-site examination of echocardiogram. It is worth noting that several members from this pedigree (II-4, III-3, III-5, and III-7) died of heart disease according to the information provided by the proband's father.

## The Pedigree Is Not Associated With Hemochromatosis

Given that a similar chromosomal inversion in chromosome 6 <inv (6) (p21.1p23)> was reported, linking to hereditary hemochromatosis with potential cardiomegaly (15), blood samples from six members in the pedigree, including four mutation carriers, were examined. Iron in the serum of the proband's father and grandmother was found to be increased, with no other abnormality found (Table 1). Their cardiac function was also examined, and no abnormality was shown in the echocardiogram (Table 2). No cardiac disease was diagnosed in a routine cardiac examination for these family members.

**Table 1:** Blood examination of members in the pedigree detecting hemochromatosis.

	**Mother** **(IV-4, 34 years old)**	**Father** **(IV-3, 44 years old)**	**Brother** **(V-3, 6 years old)**	**Grandmother** **(III-2, 70 years old)**	**Aunt** **(IV-2, 47 years old)**	**Cousin** **(V-2, 13 years old)**
FER (ng/mL)	60.75	275.95	38.73	430.88	129.55	42.73
TIBC (g/L)	56.8	48.4	56.6	53.5	42.6	69.3
FE (umol/L)	12.2	15.1	14.3	16.7	13.6	11.8
TRF (umol/L)	2.77	2.43	2.60	2.33	2.05	3.00

*FER, total iron in serum (normal range value: 21.81–274.66 for male; 4.63–204.00 for female); TIBC, total iron binding capacity (normal range value: 40.8–76.6); FE, ferritin (normal range value: 5.8–34.5); TRF, transferrin (normal range value: 2.00–3.60)*.

**Table 2:** Echocardiogram examination of the members in the pedigree.

	**Mother** **(IV-4, 34 years old)**	**Father** **(IV-3, 44 years old)**	**Brother** **(V-3, 6 years old)**	**Grandmother** **(III-2, 70 years old)**	**Aunt** **(IV-2, 47 years old)**	**Cousin** **(V-2, 13 years old)**
Aortic valve (mm)	27	29	19	26	28	27
Left atrium (mm)	29	33	23	28	31	27
Right atrium (mm*mm)	47*38	44*40	32*27	42*33	46*37	44*34
LVIDd (mm)	45	48	32	41	40	47
IVSd (mm)	11	11	7	9	9	9
LVPWd (mm)	8	7	6	8	7	9
The left ventricular wall motion	Normal	Normal	Normal	Normal	Normal	Normal
TDI (cm/s)	14	10	17	9	13	16
Ejection fraction (%)	70	74	65	78	71	65

Data were obtained from a routine transthoracic echocardiogram. Cardiac function is normal for all individuals without sign of cardiomegaly.

*LVIDd, left ventricular internal dimension—diastole; IVSd, interventricular septal thickness—diastole; LVPWd, left ventricular posterior wall thickness—diastole; TDI, tissue Doppler imaging*.

## Discussion

In this study, a mutation of chromosomal inversion in chromosomal 6 is identified to be associated with multiple congenital heart defects, including VSD and atrial septal defect. The enlargement of the septal defect in the heart (double from 0.48 to 1.04 cm) of the patient is more significant than the growth of her body, indicating that the defect does not tend to grow on its own after birth, and it is more likely to become larger, which leads to increased shunting and the progression of pulmonary hypertension that significantly affect the patient's heart function. Therefore, cardiac surgery is required in this case. The possession of the same type of congenital heart defect and, most likely, the same genetic mutation by the proband (V-4), her cousin (V-1), and her aunt (IV-5) in this pedigree indicates that chromosomal rearrangement is a major factor for the pathogenesis of VSD. However, the proband's father (IV-3), aunt (IV-2), elder brother (V-3), and cousin (V-2) carry the same mutation but with lack of an obvious congenital heart defect. There could be several possibilities: (1) these individuals, carrying the genetic mutation, have a less severe VSD that grew itself after birth and (2) other factors, such as environmental factors or genetic modifiers, contributing to the VSD are missing; therefore, no developmental cardiac defect is formed in these mutation carriers.

The human chromosomal fragile site at 6p23 is associated with multiple chromosomal rearrangements, which lead to congenital disorders. For example, the chromosomal translocation < t(6;9)(p23;q34)> often links to acute myeloid leukemia due to the generation of chimera genes in the translocation event (16, 17). A balanced translocation t(6;9)(p23;q22.3) is tightly associated with orofacial clefting (18). Endothelin 1 in Chr 6p23 is indicated to have a significant linkage with the orofacial cleft defects (18, 19). It is worth noting that a similar balanced paracentric inversion of chromosome 6, inv(6)(p21.1p23), is reported to be associated with hereditary hemochromatosis (HFE) (15). The locus of HFE has been further shown by linkage analysis to localize on the short arm of chromosome 6, adjacent to the major histocompatibility complex (15). While the examination excluded HFE in this reported pedigree, the indicated gene structure alteration involved in chromosomal inversion between these two pedigrees are different.

Although VSD has been shown often as caused by chromosomal abnormalities, to the best of our knowledge, the current study is the first report to link VSD to chromosomal abnormalities associated with the fragile site at 6p23, which may represent a new genetic etiology for VSD. However, the lack of identifying gene/locus, responsible for VSD, in the chromosomal abnormality is the limitation of the current study. Interestingly, several genes that reside in the proximal regions of these breakpoints, including TNXB (20) and CDKN1A (21) at around 6p21.3 and TFAP2A (22), EDN1 (23), and JARID2 (24) at around 6p23, were reported to be involved in the pathogenesis of VSD. Furthermore, duplication of the TNXB locus was considered as pathogenic to pulmonary atresia with ventricular septal defect in the human patient (25). In the future, the coverage of this genetic mutation in VSD and the major linked gene(s) in this chromosomal inversion need to be further determined in detail.

## Data Availability Statement

The datasets presented in this study can be found in online repositories. The names of the repository/repositories and accession number(s) can be found in the article/supplementary material.

## Ethics Statement

The studies involving human participants were reviewed and approved by Medical ethics committee of the First Affiliated Hospital, Sun Yat-sen University. Written informed consent to participate in this study was provided by the participants' legal guardian/next of kin. Written informed consent was obtained from the individual(s), and minor(s)' legal guardian/next of kin, for the publication of any potentially identifiable images or data included in this article.

## Author Contributions

LC, YT, and Z-PH planned the manuscript. LC and YT collected and analyzed the clinical data. YL and HB collected information and samples from members of the pedigree. YD performed echocardiogram for members of the pedigree. DC and ML performed karyotype examination and blood examination for members of the pedigree. Z-PH drafted the final version of the manuscript. PP and YQ revised the manuscript. All authors read and approved the final manuscript.

## Conflict of Interest

The authors declare that the research was conducted in the absence of any commercial or financial relationships that could be construed as a potential conflict of interest.
